# DNA replication inhibitor hydroxyurea alters Fe-S centers by producing reactive oxygen species *in vivo*

**DOI:** 10.1038/srep29361

**Published:** 2016-07-11

**Authors:** Meng-Er Huang, Céline Facca, Zakaria Fatmi, Dorothée Baïlle, Safia Bénakli, Laurence Vernis

**Affiliations:** 1 Institut Curie, PSL Research University, CNRS UMR3348, 91405 Orsay, France; 2 Institut Curie, CNRS UMR2027, 91405 Orsay, France; 3 Université Paris Sud, Université Paris-Saclay, CNRS UMR3348, 91405 Orsay, France

## Abstract

Redox homeostasis is tightly controlled in cells as it is critical for most cellular functions. Iron-Sulfur centers (Fe-S) are metallic cofactors with electronic properties that are associated with proteins and allow fine redox tuning. Following the observation that altered Fe-S biosynthesis is correlated with a high sensitivity to hydroxyurea (HU), a potent DNA replication blocking agent, we identified that oxidative stress response pathway under the control of the main regulator Yap1 attenuates HU deleterious effects, as it significantly increases resistance to HU, Fe-S biosynthesis and DNA replication kinetics in the presence of HU. Yap1 effect is mediated at least in part through up-regulation of two highly conserved genes controlling cytosolic Fe-S biosynthesis and oxidative stress, Dre2 and Tah18. We next observed that HU produces deleterious effects on cytosolic Fe-S clusters in proteins *in vivo* but not *in vitro*, suggesting that HU’s impact on Fe-S *in vivo* is mediated by cellular metabolism. Finally, we evidenced that HU exposure was accompanied by production of reactive oxygen species intracellularly. Altogether, this study provides mechanistic insight on the initial observation that mutants with altered Fe-S biosynthesis are highly sensitive to HU and uncovers a novel mechanism of action of this widely used DNA replication inhibitor.

Hydroxyurea (HU) is a chemical compound first synthesized in 1869 in Germany by Dressler and Stein, but which antiproliferative activity was only reported in the 1960–70’s^1^,^2^,^3^. Early studies had also shown HU impact on nucleosides incorporation^4^,^5^ mainly attributed the inactivation of ribonucleotide reductase (RNR)^6^,^7^,^8^. Indeed HU can scavenge the tyrosyl radical in the R2 subunit of RNR^9^, which is essential for enzyme activity and is otherwise very stable^10^. Because the radical mechanism is conserved among different RNRs, HU proved to be active in many organisms. HU is still widely used in clinics for treating several disorders such as chronic myeloid leukemia, sickle cell disease, AIDS and others. If HU’s DNA replication blocking properties are easily related to antitumor activity which is of benefit in myeloid leukemia and HIV proliferation, it is not so clear how HU can help against sickle cell anemia. Noteworthy, HU treatment provokes a boost in the levels of fetal hemoglobin (Hb F,α_2_γ_2_) possibly due to nitric oxide production^11^. HU is also very commonly used for cell cycle studies, as it efficiently and reversibly blocks DNA replication. In accordance, cells with mutated alleles involved in DNA metabolism often turn out to be sensitive to HU. In contrast, many genetic screening based on HU hypersensitivity identified players that are not always directly related to DNA metabolism^12^,^13^.

Dre2-Tah18 is a protein complex that has been identified in yeast by different methods^14^,^15^,^16^. Tah18 is a diflavin oxido-reductase, exhibiting three redox domains: a flavodoxin-like domain binding flavin mononucleotide (FMN), a flavin adenine dinucleotide (FAD) and a nicotinamide adenine dinucleotide (NAD) binding domains; this organization is reminiscent of P450 reductases^16^,^17^. N-terminus of Tah18 including FMN- and FAD-binding domains, is able to bind C-terminus of Dre2 *in vivo* and *in vitro*, and this interaction is essential for yeast viability^17^. Dre2 C-terminus is highly conserved between all species except bacteria, as identified by sequence alignment studies^16^,^18^. When exogenously expressed in bacteria, Dre2 was shown to include one Iron-Sulfur (Fe-S) cluster^18^ and possibly two. *In vitro*, Tah18 is capable of reducing the Fe-S cluster in Dre2, which controls downstream synthesis of Fe-S clusters in cytosolic proteins^19^.

Studies on Dre2-Tah18 physiological role are pretty recent; the complex is implicated in cytosolic Fe-S-containing proteins biosynthesis^18^,^19^, in controlling hydrogen peroxide (H_2_O_2_)-induced cell death^16^ and in ribonucleotide reductase metallocofactor assembly^20^. Interestingly, Fe-S clusters were identified in B-type DNA polymerases that include Pol3 in yeast^21^, showing that functional DNA replication is indeed conditioned by cytosolic Fe-S biogenesis, as initially suggested by genetic data^22^. In the absence of stress, Dre2-Tah18 complex is cytosolic. After exposure of the cells to lethal doses of H_2_O_2_, the complex dissociates and Tah18-dependent mitochondria damages and cell death occur^16^. The complex was suggested to act as a biosensor for intracellular oxidative stress, promoting cell death when the redox balance is severely affected, a role in opposition to its essential function for survival in the absence of stress^16^.

Several *tah18* mutants were generated in our laboratory among which the previously described *tah18-5I5* mutant^16^. Mutated cells are not affected for DNA repair after camptothecin or gamma rays exposure^16^, but Fe-S biosynthesis is decreased^23^ and growth is severely impaired after chronic exposure to HU. These phenotypes are in accordance with a role for Tah18 in DNA replication, as also suggested by genetic links between Tah18 and Pol3 that encodes DNA polymerase delta^22^, and the presence of Fe-S clusters in Pol3^21^. In order to identify new regulators of the Dre2-Tah18 complex, we performed a multicopy suppressor screen for genes that suppress *tah18-5I5* sensitivity to HU when overexpressed. Two genes, *YAP1* and *DRE2* were identified, and their roles in HU resistance are studied through this work.

Dre2 physically interacts with Tah18 *in vivo*^16^ and this interaction is essential for yeast viability^17^. However, no link between *YAP1* and *TAH18* had been anticipated previously. *YAP1* is a transcription factor that has been identified in yeast on the basis of its sequence homology with human AP-1 transcription factor^24^,^25^ and is considered as a key player in regulating the response to oxidative stress^26^, activating the transcription of a whole set of genes encoding antioxidant activities called the “Yap1 regulon”^27^. In response to H_2_O_2_ treatment, activation of transcription by Yap1 overlaps with activation by another transcription factor, Skn7, which allows fine tuning of the response to oxidative stress^27^,^28^. Transcription activating activity of Yap1 is regulated by its nuclear localization. In the absence of stress, Yap1 operates a continuous cycle between cytosolic and nuclear compartments, due to the dual presence of a nuclear localization signal (NLS) in its N-terminal region and a nuclear export signal (NES) in its C-terminal region^29^,^30^. In unstressed conditions, Yap1 is primarily located in the cytoplasm. In the presence of oxidative stress such as H_2_O_2_, Gpx3 catalyzes an intramolecular disulfide bond between the redox-sensitive cysteines of Yap1, which in turn prevents the binding of the exportin Crm1 to Yap1 C-terminus. As a consequence, Yap1 is not exported efficiently in the cytosol and accumulates in the nucleus^30^, permitting increased transcription of Yap1-controlled genes. In such oxidizing conditions, Yap1 degradation is also accelerated, restricting the duration of Yap1 effect in response to oxidative stress^31^. Yap1 was also suggested to be reduced by thioredoxins as the absence of thioredoxins provokes constitutive Yap1 oxidation^32^,^33^.

The present study reports an unexpected genetic interaction between *YAP1* and *TAH18* in the presence of HU. We identified that HU alters Fe-S centers *in vivo* through the production of reactive oxygen species (ROS). We further demonstrate that *YAP1* alleviates yeast sensitivity to HU and DNA replication kinetics in the presence HU both by decreasing ROS and improving Fe-S biosynthesis.

## Results

### *YAP1* or *DRE2* overexpression improves *tah18-5I5* mutant’s growth in the presence of hydroxyurea

**Figure 1:**
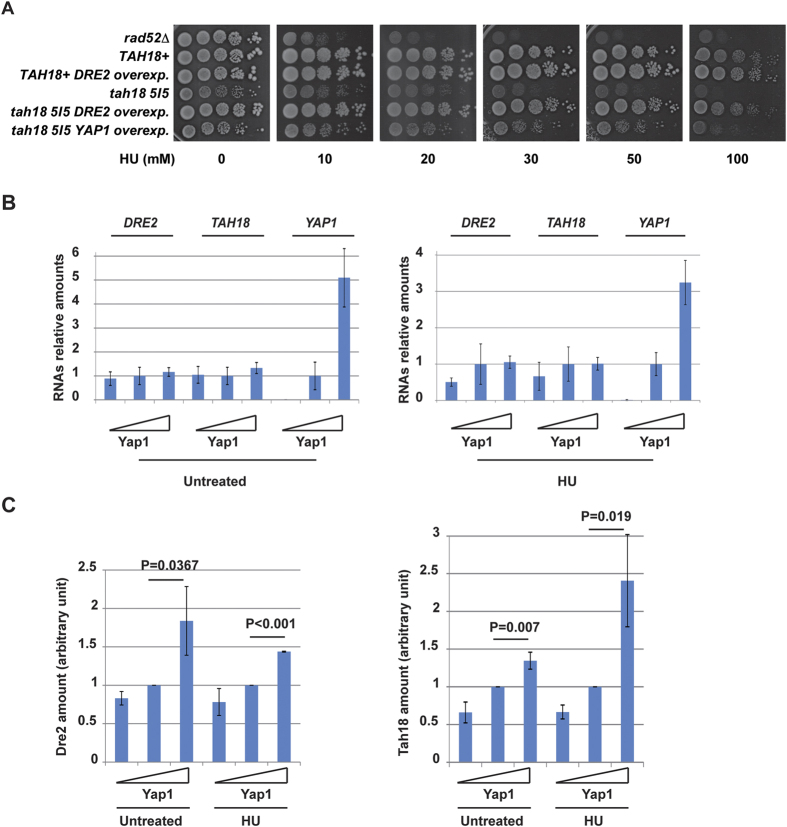
Tah18 and Dre2 are regulated by Yap1. (**A**) *DRE2* and *YAP1* overexpression can rescue viability of *tah18* cells upon HU chronic exposure. 10-fold serial dilutions of yeast cells grown until log phase were spotted on YPD medium containing increasing concentration of HU as indicated. Growth was assessed after 4 days at 28 °C. (**B**) Average expression of *DRE2* and *TAH18* mRNA levels in three strains with increasing *YAP1* expression levels. mRNA levels have been quantified in three strains: no *YAP1* expression (*yap1*Δ), wild-type *YAP1* expression (WT) and *YAP1* overexpression (*YAP1* carried out on a multicopy plasmid). Increasing expression of *YAP1* in the three strains is illustrated as a triangle. mRNA level of each gene was calculated as ratio relative to that of the wild-type (WT) strain (set as 1). (**C**) Dre2 and Tah18 protein levels in three strains with increasing *YAP1* expression levels. Yeast cells were treated with 100 mM HU for 1 hour and collected for protein analysis. Dre2 and Tah18 were detected with specific antibodies and actin was used as an internal control. Odyssey infrared imaging system was used for quantification. Relative Dre2 or Tah18 amounts were calculated as follow: Dre2 or Tah18 amounts = [Dre2 or Tah18 band intensity]/[Act1 band intensity]. Amounts in WT were set as 1. The reported values are the means of three independent experiments ± SEM. P values were calculated using unpaired one-tailed t-test, n = 3.

A *tah18* mutated allele, namely *tah18-5I5*, was generated previously using mutagenic PCR^16^ and contains several point mutations. This allele confers impaired growth over 32 °C to the yeast cells. This allele did not confer any particular sensitivity when yeasts were exposed to the DNA-damaging agent camptothecin, creating lesions in a topoisomerase I-dependent manner, nor to gamma rays that generates DNA lesions mainly in the form of base damage and double and single strand breaks^16^. In contrast, *tah18-5I5* mutant revealed to be highly sensitive to chronic exposure to HU, as described also by other authors^20^. Growth was strongly inhibited from 20 mM for both *tah18-5I5* mutant and the *rad52*Δ mutant (control), known as very sensitive to this drug (Fig. 1A). We performed a multicopy suppressor screen to identify suppressor genes of *tah18-5I5* mutant sensitivity to HU, as this might also help to understand the link of Tah18 with DNA replication. *Tah18-5I5* mutant was transformed by a multicopy 2 μ-based yeast DNA library constructed in YEp13 vector (*LEU2* marker) and cells were plated SC–LEU medium at 28 °C to select yeasts containing plasmids. Transformed cells were then replica plated on 50 mM HU-containing plates at 28 °C and growing clones were selected. Plasmids were retrieved and tested by re-transformation of the *tah18-5I5* mutant strain. Finally, DNA sequencing revealed that two genes, *DRE2* and *YAP1*, were able to suppress *tah18-5I5* sensitivity to HU when overexpressed (Fig. 1A).

Identifying *DRE2* as a multicopy suppressor of *tah18-5I5* could be reasonably expected, given that *DRE2* overexpression is known to stabilise the Dre2-Tah18 complex by mass action and to suppress *tah18-5I5* mutant thermosensitive growth^16^. In contrast, functional links between *TAH18* and *YAP1* had not been previously anticipated. Identification of *DRE2* and *YAP1* through this new screen indicates that the Dre2-Tah18 complex plays a role in response to HU chronic exposure, and that oxidative stress response might be involved in this process as well.

### *YAP1* overexpression increases Tah18 and Dre2 levels

Yap1 has been extensively studied as a transcription factor required for oxidative stress response (for review see^26^,^34^). We thus assessed whether Yap1 may regulate the transcription of *TAH18* or/and *DRE2* in the absence of stress as both proteins form a highly stable cytosolic complex^16^. In order to mimic increasing levels of Yap1 *in vivo*, we used a set of yeast strains that are respectively deleted for *yap1 (yap1*Δ), expressing *YAP1* at the endogenous level (*YAP1*), or overexpressing *YAP1* from a multicopy plasmid (*YAP1 overexp*.). These three strains were challenged with HU and RNAs were quantified. Quantitative RT-PCR analysis indicated that absence, presence or overexpression of *YAP1* had little effect on *TAH18* and *DRE2* mRNA levels in treated or untreated cells (Fig. 1B). Challenging cells with HU did not change this result. We next quantified Dre2 and Tah18 intracellular protein amounts using western blotting and polyclonal antibodies against Dre2 and Tah18 that can recognize their target with a high specificity (Ref. 16 and Supplementary. Fig. 1). Dre2 and Tah18 amounts increased as Yap1 levels increased (Fig. 1C), independently of the presence or absence of HU, showing that Yap1 levels influences Dre2 and Tah18 levels *in vivo* in a HU-independent manner. Taking these data together, we conclude that Tah18 and Dre2 levels are controlled by Yap1 *in vivo* to a moderate extent, as *YAP1* overexpression results in a subtle increase in the amount of Dre2-Tah18 complex. Thus, part of Yap1 activity in HU resistance might occur through increasing amounts of Dre2-Tah18 complex. These results might also indicate that Yap1, by decreasing oxidative stress, protects Dre2 and Tah18 from degradation and stabilizes the complex.

### *YAP1* overexpression improves cytosolic Fe-S proteins synthesis and release from hydroxyurea block in *tah18* cells

**Figure 2:**
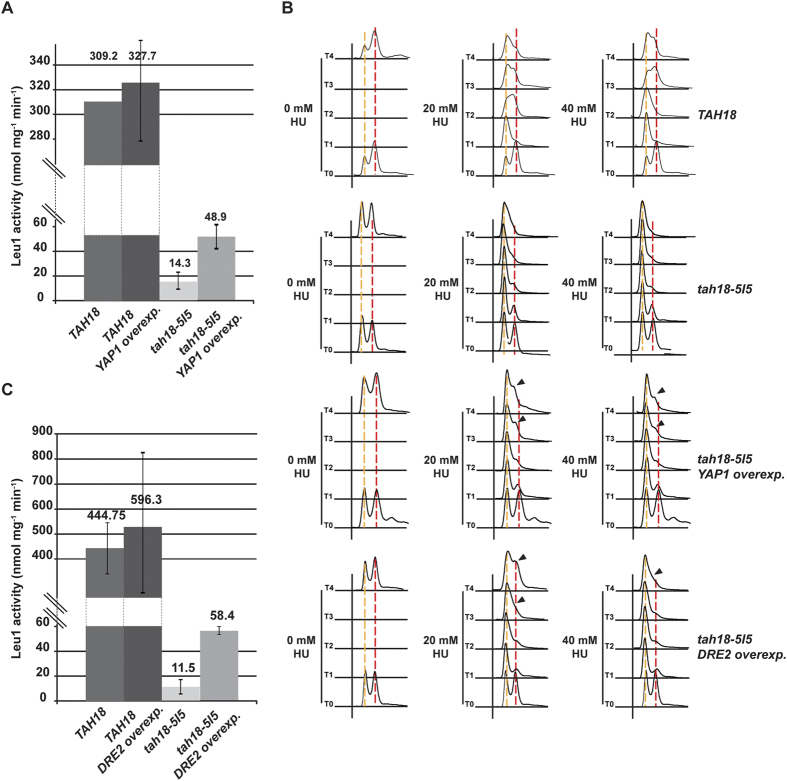
*YAP1* or *DRE2* overexpression improves *tah18-5I5* cytosolic Fe-S biosynthesis and hydroxyurea block release. (**A,C**) Isopropylmalate isomerase (Leu1) activity measurement. Citraconate was used as a substrate of Leu1. Leu1 activity (nmol mg^−1^ min^−1^) was calculated from the decrease in OD at 235 nm due to citraconate isomerization. The genotype of yeast strains used is indicated. The reported values are the means of three independent experiments ± SEM. (**B**) Flow cytometry analysis of yeast cells incubated in the presence of HU (20 and 40 mM as indicated). The genotype of yeast strains used is indicated. Time points at 0, 1, 2, 3 and 4 hour exposure are shown. S phase restart is indicated by an arrow in *tah18-5I5* cells overexpressing *YAP1* or *DRE2*.

Consistent with its role in Fe-S biogenesis, *tah18-5I5* mutant exhibits decreased cytosolic Fe-S proteins biosynthesis^17^,^19^. To test the hypothesis that overexpressing *YAP1* can improve Dre-Tah18 activity, we next examined whether overexpressing *YAP1* can rescue decreased cytosolic Fe-S biosynthesis in *tah18* cells. We measured the activity of Leu1, a cytosolic isopropylmalate isomerase containing Fe-S, which catalyzes the second step in the leucine biosynthesis pathway. Its activity can be measured by monitoring the consumption of the artificial substrate of Leu1, citraconate, and is considered as a good reporter of cytosolic Fe-S proteins biosynthesis^35^. As previously noted^17^, Leu1 activity was strongly reduced in *tah18-5I5* mutants but found indeed significantly increased in presence of overexpressed *YAP1*, reflecting an improved cytosolic Fe-S biosynthesis (Fig. 2A).

HU is a chemical compound known to scavenge tyrosyl free radicals in RNR; as a consequence, it decreases deoxyribonucleotides (dNTPs) biosynthesis and inhibits DNA replication^36^. In accordance, cells exposed to HU block their cell cycle at the very beginning of S phase before progressively returning to cycling after adapting to the drug. We examined whether *YAP1* overexpression might influence cell cycle arrest in the presence of HU. Cells were subjected to low doses of HU (20 and 40 mM) and consequently blocked their cell cycle after 2 hours as shown by the G1-S pick (Fig. 2B). Kinetics in *tah18-5I5* cells is slower than that usually observed in wild-type cells, and is actually consistent with slow growth phenotype of *tah18-5I5* cells (doubling time >3 hours). After 3 hours in the presence of HU, *tah18-5I5* overexpressing *YAP1* escaped the block as S phase cells were visible, in contrast to control *tah18-5I5*, indicating that *YAP1* overexpression permits the cells to bypass HU-induced cell cycle arrest (Fig. 2B). Therefore, activation of the oxidative stress response by overexpressing *YAP1* in *tah18-5I5* mutant improves cytosolic Fe-S cluster biosynthesis and release from HU-induced cell cycle arrest.

### *DRE2* overexpression also improves cytosolic Fe-S cluster biosynthesis and release from hydroxyurea block in *tah18* cells

As described above, we observed that *DRE2* overexpression improves growth of *tah18-5I5* mutants in the presence of HU (Fig. 1A). We verified whether overexpressing *DRE2* also rescues decreased cytosolic Fe-S biosynthesis in *tah18* cells.

As found with *YAP1* overexpressed, Leu1 activity was partially restored in case of *DRE2* overexpression, indicating that *DRE2* overexpression improves cytosolic Fe-S cluster biosynthesis (Fig. 2C). We also analysed whether *DRE2* overexpression influences DNA replication by measuring kinetics of yeast cells release from an HU block. *tah18-5I5* cells were grown in presence of mild doses of HU, as previously described. *DRE2* overexpression in *tah18-5I5* cells allowed bypassing HU effects (Fig. 2B), as an early exit from the block is visible from 3 and 4 hours after the block. Taken these observations together, we conclude that *DRE2* overexpression significantly improves cytosolic Fe-S biosynthesis and release from HU block in *tah18-5I5* cells, comparable to *YAP1* overexpression. These results indicate that improving Fe-S biosynthesis is of benefit for yeast cells in the presence of HU.

### Hydroxyurea decreases Fe-S levels in yeast cells but not in purified Leu1

**Figure 3:**
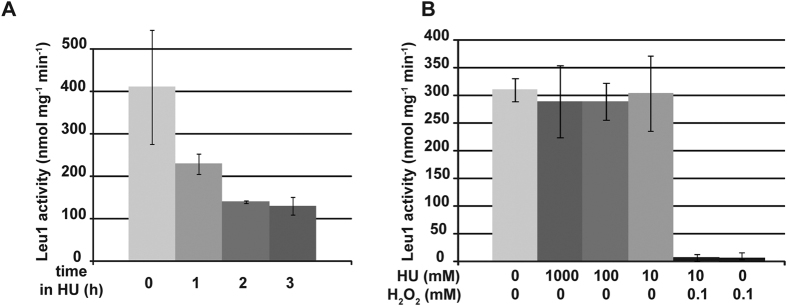
Hydroxyurea targets Fe-S centers *in vivo*. (**A**) Leu1 activity measurement of semi-purified protein extract from cells exposed to 200 mM HU for 1, 2 and 3 hours. (**B**) Leu1 activity measurement of semi-purified protein extract in the presence of HU and/or H_2_O_2_. HU and/or H_2_O_2_ have been added *in vitro* to partially purified protein extract as indicated, and activity was measured. The reported values are the means of three independent experiments ± SEM.

HU is well known for inhibiting dNTPs synthesis by scavenging tyrosyl free radicals involved in NDPs reduction into dNDPs^37^,^38^. As a consequence, HU has been mainly regarded as a DNA replication blocking agent, but less as a redox-interfering drug. Because *tah18-5I5* mutant (this work) and a *dre2* mutant (not shown) are highly sensitive to HU, and because increasing cytosolic Fe-S biosynthesis improves yeast resistance to HU, we examined whether HU might have deleterious effects on cytosolic Fe-S. Wild-type yeast cells were incubated in the presence of HU for 1, 2 and 3 hours, before being collected and assessed for cytosolic Fe-S level using Leu1 assay. In the presence of HU, Leu1 activity was found significantly decreased by 3- to 4-fold, showing that indeed cytosolic Fe-S are altered in cells that have been cultivated in the presence of HU (Fig. 3A).

We next assessed whether HU acts directly on Fe-S clusters. Semi-purified Leu1 extracts from untreated cells were prepared, and HU was then added directly into the extracts before assessing Leu1 activity. H_2_O_2_ was used as a control, as it directly oxidizes Fe-S^39^. As shown in Fig. 3B, direct addition of HU within the extracts did not affect Leu1 activity even using high HU concentration (1 M as compared to 200 mM classically used in yeast cells media). In contrast, semi-purified Leu1 retained no activity in the presence of micromolar amounts of H_2_O_2_ (Fig. 3B), arguing that deleterious effects of HU on Fe-S centers in yeast cells are not due to direct interaction between HU and Fe-S centers. We conclude that, different to H_2_O_2_, HU is not capable of altering Fe-S centers directly and that the previously characterized, deleterious consequences of HU on Fe-S centers might depend on cell metabolism.

### Detection of H_2_O_2_ in yeast cells exposed to hydroxyurea using sensitive redox sensor HyPer

**Figure 4:**
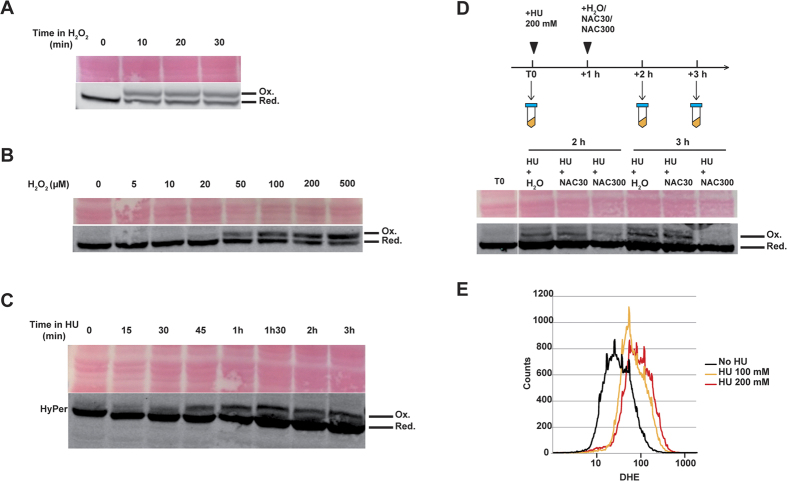
Hydrogen peroxide is detected in yeast cells after hydroxyurea exposure. (**A–D**) HyPer detection using redox western blotting. (**A**) Exponentially growing yeast cells expressing HyPer cultured in YPD were exposed to H_2_O_2_ (1 mM) for increasing times as indicated. Oxidized (Ox.) and reduced (Red.) HyPer bands are indicated, and Ponceau red staining is shown as loading control. (**B**) Yeast cells were treated for 20 min with increasing H_2_O_2_ concentrations as indicated. (**C**) Yeast cells were treated with HU (200 mM) for increasing times as indicated. (**D**) Yeast cells were treated with HU (200 mM) for 1 hour. NAC (30 mM or 300 mM) was then added to cultures for 1 hour and 2 hours. H_2_O was also used as a control. Samples were processed at various times as indicated. (**E**) ROS detection using DHE. Exponentially growing yeast cells cultured in YPD were exposed to HU (100 and 200 mM) for 1 hour, before staining with DHE to evidence ROS production.

Because activating the oxidative stress response through Yap1 relieves HU phenotypes, we hypothesised that ROS could be generated in the presence of HU. Detecting low level of ROS *in vivo* is technically challenging but recently developed, genetically encoded biosensors provide an alternative way to overcome the limitations of conventional redox measurements^40^. HyPer is a genetically encoded fluorescent sensor that consists of circularly permuted yellow fluorescent protein (cpYFP) inserted into the regulatory domain of prokaryotic H_2_O_2_-sensing protein OxyR^41^. HyPer is capable of detecting production and local bursts of H_2_O_2_ in the cytoplasm and mitochondria of HeLa cells after various stimulations such as Apo2L/TRAIL treatment or in the cytoplasm of PC-12 cells stimulated by nerve growth factor^41^. HyPer construct was designed similarly to previously published redox-sensitive YFP (rxYFP) as a sensor, and stably integrated within yeast chromosomes to guarantee comparable expression between yeast cells in culture (see Methods and^42^). Redox western blot conditions were set up so that oxidized and reduced forms of HyPer could be separated on SDS-PAGE, and detected using an anti-GFP antibody. Cells were exposed to 1 mM H_2_O_2_ for increasing times (10, 20 and 30 min) to evaluate HyPer’s response time to treatment (Fig. 4A). An oxidized band appeared as soon as 10 min after bolus addition indicating fast kinetics of H_2_O_2_ detection by the probes. Finally we evaluated H_2_O_2_ detection threshold by treating cells with increasing concentrations of exogenous H_2_O_2_ for 20 min. Oxidized forms of HyPer were detected from the 20–50 μM range and over (Fig. 4B), making HyPer a sensitive tool to detect subtle H_2_O_2_ changes within yeast cells.

Yeast cells were treated with HU 200 mM for increasing times, and redox state of HyPer was monitored. An extra band appeared 30–45 min after treatment, with comparable size to oxidized HyPer (Fig. 4C), suggesting that HyPer is oxidized in response to HU. To further assess HyPer modification, the antioxidant N-Acetyl-Cysteine (NAC) was used. Yeast cells were treated for 1 hour with HU 200 mM, before adding NAC to the medium for an additional one or two hours (Fig. 4D). The intensity of the modified HyPer band decreased after treatment with NAC 300 mM for 1 hour, and the band totally disappeared after two hours, in contrast to water- or NAC 30 mM- treated samples. This confirmed that HU indeed provokes HyPer oxidation *in vivo*.

Increased ROS level following HU treatment was further evidenced using dihydroethidium (DHE). In the presence of ROS, DHE is rapidly oxidized to form a red fluorescent 2-hydroxyethidium detectable by flow cytometry (FACS). Yeast cells were treated with HU 100 mM or HU 200 mM for 1 hour and then stained by DHE for 2 hours. FACS analysis revealed increased fluorescence in the treated samples (Fig. 4E), indicative of ROS production in yeast cells after HU treatment. Taken together, these experiments show that ROS, including H_2_O_2_, are generated after HU treatment *in vivo*.

## Discussion

**Figure 5:**
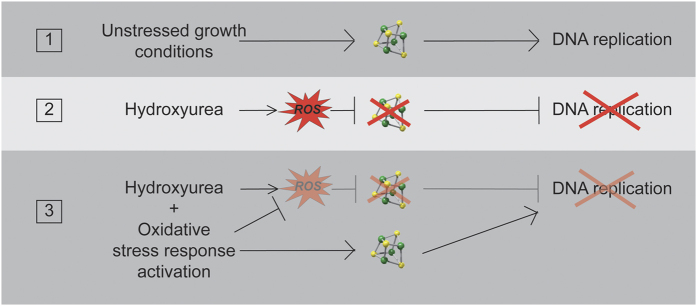
Hydroxyurea induces DNA replication arrest by producing ROS and targeting Fe-S centers. The model presented here does not show the known effects of HU on RNR. HU targets Fe-S centers *in vivo* by producing ROS, which delay DNA replication (1–2). Activation of Yap1 decreases ROS levels and increases Fe-S biosynthesis, which in turn attenuates HU-induced Fe-S alteration and improves DNA replication (3).

Thanks to a genetic screen for multicopy suppressors we identified that oxidative stress response under the control of Yap1 improves HU resistance of yeast cells that are defective for Fe-S biosynthesis (*tah18-5I5* mutant). We further demonstrate that *YAP1* overexpression actually improves Fe-S biosynthesis and release from an HU block, and that this occurs at least in part through stabilizing the Dre2-Tah18 complex. Finally, we demonstrate that HU *per se* alters Fe-S centers *in vivo* through a mechanism that involves the production of ROS. This sheds light on the role of oxidative stress response in the resistance to HU. As summarized in Fig. 5, we propose that Yap1 acts in HU resistance by different means. First, it is involved in eliminating ROS that are produced during HU exposure due to its transcriptional activity controlling numerous antioxidant proteins. *TRX2* encodes a cytosolic thioredoxin that is a key player in the response to oxidative stress, and which expression is finely regulated by Yap1^43^. Overexpressing *TRX2* slightly improved *tah18-5I5* mutants’ growth on HU (Supplementary Fig. 2) in accordance with the fact that increased antioxidant activity is of benefit during HU exposure. This result also suggests that at least part of Yap1 effect in HU resistance is mediated by Trx2. Second, Yap1 increases Dre2-Tah18 complex, which improves Fe-S biogenesis and thus counteracts Fe-S alteration in the presence of HU. This result is consistent with previous data showing that Dre2, as an Fe-S-containing protein, is highly sensitive to oxidation *in vitro*^17^. It is thus possible that Dre2 is sensitive to oxidative stress also *in vivo*, so that *YAP1* overexpression, by slightly increasing Dre2 levels, stabilizes Dre2-Tah18 complex. It is interesting that, in addition to ROS production, HU might also perturb Fe-S formation by scavenging tyrosyl radicals of proteins involved in Fe-S biosynthesis. This could then decrease even more Fe-S levels *in vivo*.

It is well documented, particularly in bacteria, that many Fe-S centers are sensitive to oxidative agents. Fe-S proteins are inactivated by superoxide anion (O_2_^•−^)^44^ and by very low levels of H_2_O_2_^39^,^45^. Mice lacking the mitochondrial superoxide dismutase *SOD2* exhibit chronically increased levels of oxidative stress (O_2_^•−^) associated with decreased activities of aconitase and succinate deshydrogenase, two Fe-S-containing enzymes^46^. In this study, we evidenced ROS production occurs after HU treatment in yeast cells, which provides mechanistic insights to the deleterious effects of HU toward Fe-S. Several arguments in literature support our finding. Notably, catalase, which eliminates H_2_O_2_ *in vivo*^47^ was identified as a target of HU in plants^12^. It is thus possible that catalase inhibition by HU participates to the H_2_O_2_ boost we evidenced. Also, respiration-defective mutants with notably reduced endogenous ROS production were found significantly more resistant to HU in bacteria. Furthermore, addition of the OH^•^ scavenger thiourea in the presence HU suppressed bacteria sensitivity to HU, suggesting that ROS generation is the direct cause of HU-induced cell death^13^. Actually it is unknown whether H_2_O_2_ is the only ROS produced in HU. O_2_^•−^ might also be produced, as indicated by DHE sensor (Fig. 4E), even though O_2_^•−^ is not a strong oxidant, but it is highly deleterious to some Fe-S centers^44^. O_2_^•−^ is rapidly converted to H_2_O_2_ either by spontaneous dismutation or by the action of superoxide dismutases (SODs)^48^. Other byproducts might also include nitroxide, as shown in rats^49^.

Fe-S centers are also targeted by metallic cations *in vitro* and *in vivo*^50^,^51^. Beside its well known property to inhibit RNR activity *in vivo*, HU is reported to interact with metallic ions such as Cu^2+^, Fe^2+^ and Fe^3+^ and form complexes, and that may affect its pharmacological properties^52^. *In vitro*, the interaction between HU and Fe^3+^ in aqueous solutions forms a HU-Fe complex which decomposes through redox processes, forming Fe^2+^, NH_4_^+^, CO_2_ and NO_2_; HU radical species H_2_N-CO-NHO^•^ are also detected in the course of the reaction^53^. HU may interfere with electron transfer processes in biological systems and might induce damage to cellular macromolecules^54^. A few studies reported that indeed HU is a Fe^3+^ reducing agent *in vivo* as well, and was even suggested to be a clinically relevant iron chelator^55^. In yeast, interplay between HU and iron mobilization has been studied^56^. Strikingly, the authors identified that the transcriptional response to HU in yeast is partially overlapping with the low iron stress response. Part of the transcriptional response to HU is dependent on the Aft1 and Aft2 transcription factors, known to control iron homeostasis in response to changes in iron availability^57^. This is consistent with results obtained in bacteria, as HU stress also activates iron uptake^58^. Altogether, these studies are in agreement with the fact that HU may act as an iron chelator in cells, as suggested above. For these reasons, we tested whether iron homeostasis might be compromised in *tah18-5I5* mutants, as this could influence sensitivity to HU. Excess iron or iron depletion did not influence *tah18-5I5* growth in the presence of HU (Supplementary Fig. 3), indicating that iron homeostasis is not severely affected and is probably not the major cause of HU sensitivity in *tah18-5I5* mutants.

HU has been used in clinics to block cell proliferation and is known to inhibit DNA replication by decreasing intracellular dNTP pool^59^. Interestingly, it is now known that Dre2-Tah8 is involved in RNR metallocofactor assembly as an electron donor, playing a critical role in the formation of Fe^3+^ 2-Y^•^ cofactor in RNR and in supplying dNTPs^20^. In accordance, synthetic lethality had been originally observed between a mutated allele of the Fe-S cluster-containing DNA polymerase delta subunit *pol3*, and *dre2* or *tah18* mutants^22^. It is thus probable that RNR is compromised in the mutated *tah18-5I5* strain; Dre2 and Tah18 increased expression resulting from *YAP1* overexpression might then also improve dNTP content and thus counteract HU effects.

Several studies pointed out that dNTPs exhaust is not complete after exposure to HU, and that basal dNTPs levels are maintained in a checkpoint-independent manner^59^,^60^. These authors suggested that the persistence of a certain dNTPs level after HU-induced DNA replication arrest might be helpful for other DNA metabolisms such as DNA repair. Independently, it is well known that numerous DNA-transacting proteins contain Fe-S centers, among which Rad3^61^, DNA primase^62^,^63^, DNA2^64^, XPD and FancJ^65^, Pol3^21^, this very last being necessary for the formation of an active replication complex, at least *in vitro.* Because Fe-S centers in Leu1 are altered when cells are exposed to HU, we speculate that Fe-S centers in Pol3 and in other Fe-S DNA replication proteins might be altered as well. This could account for the fact that HU arrests DNA replication even though the dNTP pool is not fully depleted^59^.

## Methods

### Strains and media

All strains used in this study are isogenic, except EGY48 from Clontech, 3B9 (BY4741) and 21A5 (double mutant *fet3*Δ *fet4*Δ) (Table 1). Gene disruptions were made by standard PCR-based methods. Yeast strains were grown in YPD medium (1% yeast extract, 2% bacto peptone, 2% glucose). For YPD plates, 2% agar was added. For *URA3*-containing plasmid selection, yeasts were grown on Synthetic Complete (SC) medium lacking uracil (SC–URA) (0.67% yeast nitrogen base, adenine, histidine, lysine, leucine, tyrosine, methionine, tryptophan, 40 mg/liter for each, 2% glucose, 3% agar). HU, iron citrate and bathophenantroline (BPS) were purchased from Sigma. 30 mM stock solution of BPS was prepared in ethanol. For cell cycle experiments, 20 and 40 mM HU were used in exponential yeast cultures. 1 ml of cell culture was collected at indicated time points, and processed for flow cytometry. Detailed information regarding strain construction is provided as Supplementary Methods.

### Cloning

All pRS vectors used in this study have been described^66^. Multicopy 2 μ-based yeast DNA library made in YEp13 vector (*LEU2* marker) was a kind gift from B. Daignan-Fornier. For construction of pRS303-HyPer, plasmid MEHp97^42^ was restricted using *Sac*II and *Not*I, generating an insert containing rxYFP coding sequence under the control of the PGK promoter. This insert was ligated into pR303^66^ digested by *Sac*II and *Not*I, generating plasmid pRS303-Cyto-rxYFP. Plasmid pRS303-Cyto-rxYFP was digested with *Not*I*-Xba*I, yielding a 785 bp-DNA fragment containing PGK1 promoter. DNA fragment was subcloned into pRS303, yielding pRS303-PromPGK. HyPer coding region was amplified by PCR with oligo 733 and oligo 735 containing Nuclear Localization Signal, using pHyPer-cyto vector (Evrogen) as a template. PCR product was digested with *Bam*HI*-Eco*RI before subcloning into pRS303-PromPGK, yielding pRS303-HyPer. For construction of pRS425-Trx2, Trx2 coding region was amplified by PCR with oligo 670 and oligo 671, using genomic DNA as a template. PCR product was digested with *Bam*HI*-Hind*III before subcloning into pRS425, yielding pRS425-Trx2. pRS425-Trx2 #2 and #12 are two independent constructs in *E. coli*.

### RNA purification and quantitative RT-PCR

Yeast RNAs were purified as described previously^67^. All buffers were added with 0.1% v/v diethylpyrocarbonate (DEPC) before being autoclaved. 50 mL of exponentially growing yeasts were pelleted and washed in cold water, before resuspending in TES buffer (10 mM Tris-HCl pH 7.5, 10 mM EDTA, 0.5% SDS) and adding equal volume of acid phenol. The mix was incubated during 1 hour at 65 °C, and aqueous phase was submitted to 3 consecutive phenol extractions. RNAs from aqueous phase were precipitated by centrifugation after addition of ethanol and 0.5 M sodium acetate. RNA were then washed with 70% ethanol and dried before resupending in H_2_O. RNAs were incubated in presence of RNAse-free DNAse I (New England Biolabs) according to the manufacturer’s instructions. The absence of contaminating DNA was further confirmed using classical PCR and 20-bp standard oligos. Next, cDNA were obtained using an iScript cDNA synthesis Kit (Bio-Rad). cDNA were amplified using iQ SYBR Green Supermix (Bio-Rad) and a set of primer pairs (Table 2). *ACT1* expression was used as an internal control. Quantitative RT-PCR reactions were carried out in the CFX96 Bio-Rad Thermal Cycler and mRNA relative levels were calculated using Bio-Rad CFX Manager software. mRNA levels of wild-type cells were normalized to 1 for each gene and each condition tested.

### Western blotting

Protein extracts were prepared using TCA method^68^. About 50 μg of proteins were separated on a 4–12% gradient NuPAGE Novex Bis-Tris gel with MOPS SDS buffer (Life Technologies). After transfer to a nitrocellulose ECL membrane (Amersham), Dre2 and Tah18 were probed with a rabbit anti-Dre2 antibody designed against Dre2 1–133 N-terminus peptide^16^ or a rabbit anti-Tah18 antibody (1/2500, described below), and a fluorescent secondary anti-rabbit IgG (IRDye, LI-COR). Proteins were quantified using Odyssey infrared imaging system (LI-COR) using Act1 as an internal control. Act1 was probed with a goat anti-Act1 antibody (sc-1616, Santa Cruz Biotechnology), and a fluorescent secondary anti-goat (IRDye, LI-COR). Amounts of proteins were calculated as follow: [protein band intensity]/[Act1 intensity]. Amounts in wild-type cells were set as 1.

### Antibody against Tah18

*TAH18* 1-600 (FMN binding domain) was subcloned into pET19b, and the plasmid obtained was transformed into *E. coli* BL21 strain. Overexpressed 6xHis-tagged, 1–200 N-terminus Tah18 peptide, was purified using NiNTA technology (Qiagen). Antisera were raised in rabbit (Agrobio Company, France) and tested for Tah18 detection by western blotting as shown in Supplementary Fig. 1.

### Redox western blotting

Cell extracts were prepared as for classic western blot using the TCA method^68^ until the step of TCA removal from pelleted broken cells. From there, pellets were washed three times with acetone. Dry pellets were resuspended in 50 μl of TES buffer (100 mM Tris-Cl pH 8.8, 10 mM EDTA, 1% SDS) containing 50 mM N-Ethylmaleimide (NEM). Following 1 hour incubation at 30 °C with strong agitation in the dark, insoluble protein was removed by centrifugation at 13000 g for 10 min at room temperature. Each sample containing about 30 μg of protein was prepared with NuPAGE LDS sample buffer without reductant and was separated on a 12% NuPAGE Novex Bis-Tris gel with MOPS SDS buffer (Life Technologies). After transfer to a nitrocellulose membrane, oxidized and reduced forms of HyPer were probed with anti-GFP (A-11122, Life Technologies).

### Isopropylmalate isomerase (Leu1) activity measurement

Leu1 activity was measured on semi-purified protein extract by successive ammonium sulphate precipitation following a method previously described by Kohlhaw *et al*.^35^. Cells were grown until mid-log phase and were harvested. Crude extracts were prepared in 50 mM phosphate buffer (pH 7.1) containing 50 mM NaCl and the 50% to 65% ammonium sulphate fraction was resuspended in 50 mM phosphate buffer (pH 7.1) containing 1.6 M ammonium sulphate and 30% glycerol. The artificial substrate citraconate was used to determine total Leu1 activity by monitoring consumption of citraconate at 235 nm using increasing amounts of protein. Protein concentrations were measured using a Quant-iT protein assay kit in a Qubit fluorimeter (Life Technologies). For evaluating direct effect of HU on Leu1 activity, semi-purified Leu1 was pre-incubated on ice for 60 min in presence of HU (1 M, 100 mM and 10 mM) or H_2_O_2_ (100 μM), before monitoring consumption of citraconate at 235 nm.

### Flow cytometry for cell cycle

1 ml of yeast cells culture was spined down for 5 min at maximum speed (14000 rpm) and supernatant was removed. Cells were immediately resuspended in 1 ml 75% ethanol and kept overnight at 4 °C. Fixed cells were spined down and pellet was washed in 1 ml Tris 50 mM (pH 7.5) before resuspension in 1 ml Tris 50 mM (pH 7.5) containing RNase mg/ml (Sigma). Suspensions were incubated 4 hours at 37 °C, before resuspending cells in 500 μL PBS buffer containing propidium iodide 0.05 mg/ml. Cell suspension was sonicated for 5 sec at medium power, before diluting cells at 1/10^th^ in Tris 50 mM (pH 7.5) and analysing samples on a Bekton Dickinson FACS Calibur. 50000 cells were counted for each acquisition.

### Flow cytometry for ROS detection

2 ml of yeast cells culture treated for 45 min with HU 100 mM or HU 200 mM were spined down for 5 min at maximum speed (14000 rpm) and supernatant was removed. Cells were washed three times with 2 ml PBS, and lastly resuspended in 1 ml PBS containing DHE 50 μg/ml. Suspensions were gently agitated in the dark for 1 hour. Cells were then washed again three times with PBS. 20 μL of cell suspension was diluted in 1 ml PBS and samples were analyzed on a Bekton Dickinson FACS Calibur using FL1 channel. 50000 cells were counted for each acquisition.

### Statistics

Unpaired one-tailed Student’s t-test was used for comparisons of protein levels in yeast strains (Fig. 1).

## Additional Information

**How to cite this article**: Huang, M.-E. *et al*. DNA replication inhibitor hydroxyurea alters Fe-S centers by producing reactive oxygen species *in vivo*. *Sci. Rep.* **6**, 29361; doi: 10.1038/srep29361 (2016).

## Supplementary Material

Supplementary Information

## Figures and Tables

**Table 1:** Yeast strains used in this study.

Strain	Genotype	Source
3B9	*MATa his3*Δ*1 leu2*Δ*0 met15*Δ*0 ura3*Δ*0 rad52*Δ*::KANR*	Euroscarf
5C4	*MATa ura3 leu2 LYS2 his3 dre2*Δ*::TRP1-CYH2S pRS416-DRE2-URA3*	16
5I5	*MATa his3 leu2 LYS2 trp1 ura3 cyh2R tah18-5I5*	16
8C2	*MATalpha HIS3 leu2 lys2 trp1 ura3 cyh2R*	17
8C3	*MATa his3 leu2 LYS2 trp1 ura3 cyh2R*	Gérard Faye
8C4	*MATa/alpha HIS3/his3 leu2/leu2 LYS2/lys2 trp1/trp1 ura3/ura3 cycloR/cycloR*	Gérard Faye
10F3	*MATa HIS3 leu2 lys2 trp1 ura3 cyh2R dre2::DRE2-eGFP-KANR*	This study
11B3	*MATa his3 LEU2-pRS305-TAH18Promoter-GFP-TAH18 LYS2 tah18*Δ*::TRP1-CYH2S ura3*	16
11B8	*MATalpha HIS3 leu2 lys2 trp1 ura3 cyh2R yap1*Δ*::HisG-URA3-HisG*	This study
12A6	*MATa HIS3 leu2 lys2 trp1 ura3 cyh2R dre2::DRE2-eGFP-KANR pRS425-YAP1-LEU2*	This study
12G2	*MATalpha HIS3 leu2 lys2 trp1 ura3 cyh2R tah18-5I5*	16
12G3	*MATalpha his3 leu2 LYS2 trp1 ura3 cyh2R tah18-5I5*	16
13E2	*MATalpha HIS3 leu2 lys2 ura3 cyh2R TRP1-pRS304-TEF1p-DRE2*	This study
13E5	*MATa his3 leu2 LYS2 ura3 cyh2R TRP1-pRS304-TEF1p-DRE2 dre2*Δ*::TRP1-CYH2S*	This study
13E6	*MATalpha HIS3 leu2 lys2 ura3 cyh2R TRP1-pRS304-TEF1p-DRE2 dre2*Δ*::TRP1-CYH2S*	This study
14C8	*MATa HIS3 leu2 lys2 ura3 cyh2R TRP1-pRS304 TEF1p-DRE2 dre2*Δ*::TRP1-CYH2S tah18-5I5*	This study
14D6	*MATalpha HIS3 leu2 lys2 trp1 ura3 cyh2R pRS426-YAP1-URA3*	This study
17D1	*MATa his3 leu2 LYS2 trp1 ura3 tah18-5I5 pRS425-YAP1-LEU2*	This study
18H6	*MATalpha HIS3 leu2 lys2 trp1 ura3 tah18-5I5 pRS425-TRX2-LEU2 #1*	This study
18H8	*MATalpha his3 leu2 LYS2 trp1 ura3 pRS425-TRX2-LEU2*	This study
18I1	*MATalpha his3 leu2 LYS2 trp1 ura3 tah18-5I5 pRS425-TRX2-LEU2 #2*	This study
19H4	*MATalpha his3 leu2 LYS2 trp1 ura3 cyh2R tah18-5I5 pRS426-YAP1-URA3*	This study
19I4	*MATa HIS3-pRS303-Hyper leu2 LYS2 trp1 ura3 cyh2R*	This study
21A5	*MATa ade6 can1 his3 leu2 trp1 ura3 fet3-2::HIS3 fet4-1::LEU2*	69

**Table 2:** Oligonucleotides used for quantitative RT-PCR.

forward *ACT1* #440	CTGCCGGTATTGACCAAACT
reverse *ACT1* #441	CGGTGATTTCCTTTTGCATT
forward *DRE2* #478	AATGGCCACTGAACCAAAAG
reverse *DRE2* #479	CATCGCTGGTATCCACTTGA
forward *TAH18* #482	GGTGTCGGTCTAGCACCATT
reverse *TAH18* #483	AATTTTGCCCTTACGGAACC
forward *YAP1* #480	ACACCAATCCCAGCCTACTG
reverse *YAP1* #481	GAATTGTCGTCTGGGAAAGC
