# Supplementary material for: DNA replication inhibitor hydroxyurea alters Fe-S centers by producing reactive oxygen species in vivo
Source: Sci Rep. 2016 Jul 11;6:29361. doi: 10.1038/srep29361 (PMC4942693; doi:10.1038/srep29361)
Supplement: Supplementary Information [file srep29361-s1.pdf]

Supplementary Information

DNA replication inhibitor hydroxyurea alters Fe-S centers by producing reactive oxygen species *in vivo*

Meng-Er Huang, Céline Facca, Zakaria Fatmi, Dorothée Baïlle, Safia Bénakli and Laurence Vernis

Summary

Supplementary Figure 1	p2
Supplementary Figure 2	p3
Supplementary Figure 3	p4
Supplementary Methods	p5

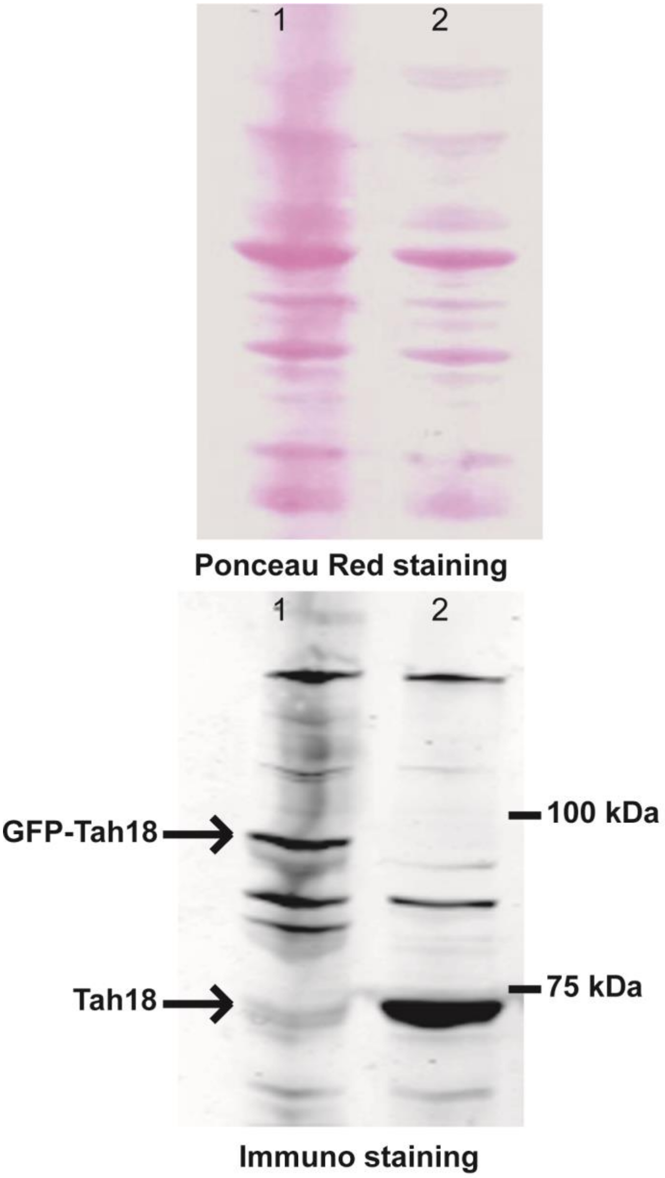

Supplementary Figure 1. Tah18 immunodetection using anti-Tah18 antiserum.

Line 1: Strain 11B3 expressing Tah18-GFP; line2: Wild-type strain 8C2. Western blotting was performed as described in the Methods section.

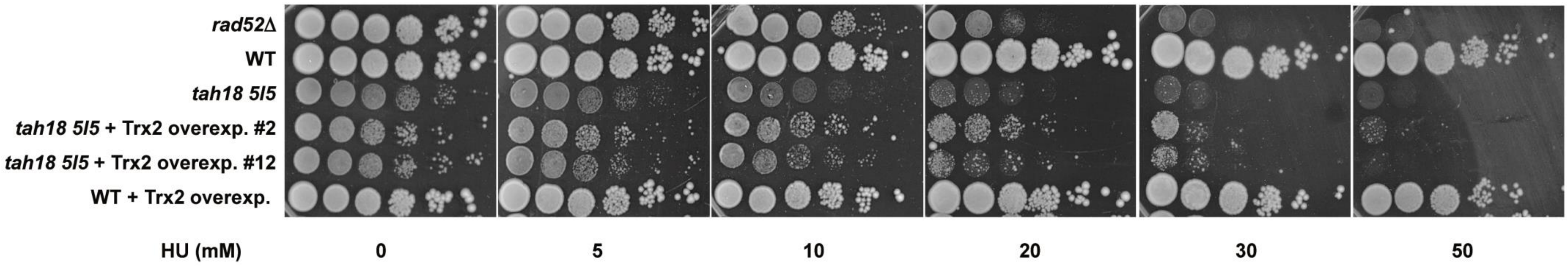

Supplementary Figure 2. Trx2 overexpression improves *tah18-5I5* yeast cells growth in presence of low doses of HU.

10-fold serial dilutions of yeast cells grown until log phase were spotted on YPD medium containing increasing concentration of HU as indicated. Growth was assessed after 4 days at 28°C.

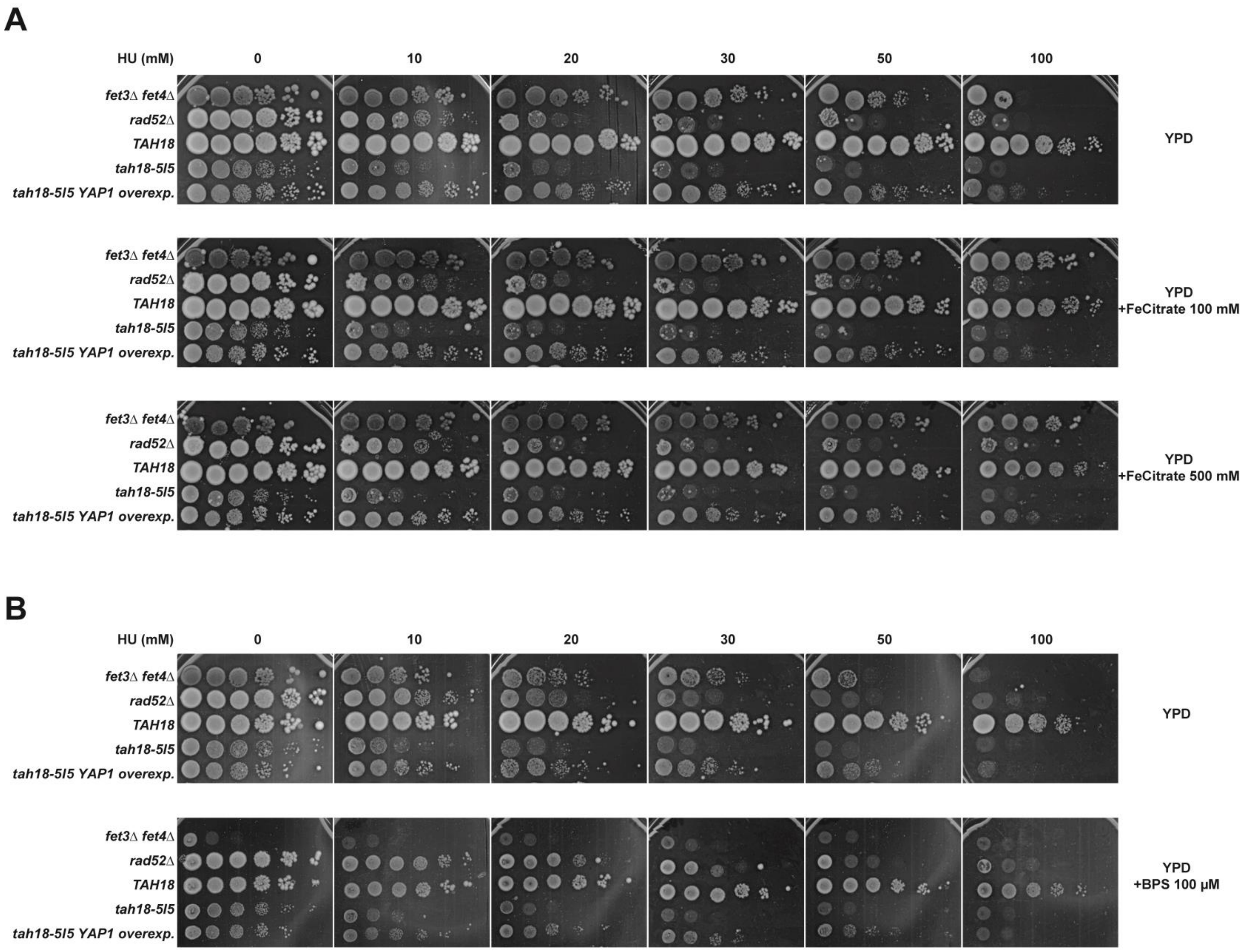

Supplementary Figure 3. Excess iron or iron depletion does not affect *tah18-5I5* yeast cells growth in presence of HU.
10-fold serial dilutions of yeast cells grown until log phase were spotted on YPD medium containing (A) excess iron (FeCitrate 100 and 500 mM) or (B) limited iron (BPS 100 µM), and increasing concentration of HU as indicated. Growth was assessed after 4 days at 28°C.

Supplementary Methods

Yeast strains construction

10F3: Enhanced GFP (eGFP) coding region was amplified by PCR with oligos 200 and 201 using pKT127 [1] as template. The resulting fragment was used to transform yeast strain 9A7, resulting in strain 10F3.

11B8: *YAP1* gene was disrupted as described in [2] using the hisG-*URA3*-hisG disruption cassette consisting of the hisG-*URA3*-hisG flanked by DNA from upstream and downstream of the YAP1 coding sequence. DNA from the upstream of *YAP1* was amplified using genomic DNA as template and primers YAP1A and YAP1B. DNA from the downstream of *YAP1* was amplified using genomic DNA as template and primers YAP1C and YAP1D. The resulting two fragments were cloned respectively at *Xba*I–*EcoR*I and *Xho*I-*Kpn*I restriction sites in plasmid p1209 [2], yielding plasmid 4B5. Plasmid 4B5 was digested with *BtsI*, and the product containing disruption cassette was used to transform diploid yeast strain 8C4. Haploid strain 11B8 was selected after sporulation of diploid transformant.

12A6: *YAP1* region including promoter was amplified using genomic DNA as template and primers 256 and 257. The resulting fragment was cloned at *Xho*I-*Pst*I in pRS425 [3] yielding plasmid 4B9. Strain 10F3 was transformed by plasmid 4B9-(*pRS425-YAP1-LEU2)*, resulting in yeast strain 12A6.

13E5, 13E6 and 14C8: TEF1 promoter was amplified using genomic DNA as template and primers TEF1Forward-*EcoR*I and TEF1Reverse-*Bam*HI. The resulting fragment was cloned at *EcoR*I-*Bam*HI in pRS304 [3], yielding plasmid 2A8. *DRE2* gene was amplified using W303 genomic DNA as template and primers 301 and 302. The resulting fragment was cloned at *Bam*HI-*Not*I in

plasmid 2A8, yielding plasmid 5D1. Plasmid 5D1 was digested with *Bsu36I* and integrated into yeast strain 8C2 at the *trp1* loci, yielding 13E2 yeast strain. 13E2 strain was then crossed with 5C4 strain containing a *dre2* deletion, and spores 13E5 and 13E6 were selected. 13E6 was crossed with 5I5 (*tah18-5I5*) and spore 14C8 was selected.

14D6: Plasmid 4B9 was digested with *Bgl*I, and *YAP1*-containing fragment was subcloned into pRS426 [3] digested with *Bgl*I. Transformation of 8C2 with this plasmid yielded yeast strain 14D6.

17D1: Yeast strain 17D1 is a spore resulting from crossing of strains 12A6 and 12G3.

18H6, 18H8 and 18I1: Strain 8C2 was crossed with strain 5I5, and diploid strain was selected and transformed by plasmid pRS425-TRX2 (#2 and #12). Haploid strains 18H6, 18H8 and 18I1 were selected after sporulation of diploid transformants.

19H4: Yeast strain 19H4 is a spore resulting from crossing of strains 12G2 and 14D6.

19I4: Plasmid pRS303-HyPer was digested with *Nhe*I and yeast 8C3 was transformed by the linearized plasmid, yielding strain 19I4.

Oligonucleotides used for plasmid and strain construction

Name	Sequence
YAP1A	GCTCTAGAGCAGTGCAAGAGGTCGCTGGATGTCG
YAP1B	CGGAATTCCGGCAAATTAGGATCTCGCCTTGC
YAP1C	CCGCTCGAGCGGCAGTAGTTTACAGAATGCTG
YAP1D	GGGGTACCCCGCAGTGAGGTGATACAATCTACCTAC
TEF1Forward-EcoRI	CGGAATTCCACACACCATAGCTTCAAAATGTTTCTA
TEF1Reverse-BamHI	CGGGATCCGTAATTAAAACTTAGATTAGATTGCTATGCTTTCTTTC
200	GGTCTTCCTGCTTTCAAGCCTGGTCAACCTATCAATTTGGACAGCAT TTCAGATGACTTGGGTGACGGTGCTGGTTTAAT

201	TTCACCTTCACCAAAGTAGACCAATTGACGTCATTTACTGAAACGA ATGTGCAGGGTTTATCGATGAATTCGAGCTCGTT
256	GCCGCTCGAGGTATTTATCGGAAACGGCAGTAAACG
257	TAACTGCAGTTAGTTCATATGCTTATTCAAAGC
301	CGCGGATCCATGTCACAATACAAAACTGG
302	ATAAGAATGCGGCCGCTTACAAGTCATCTGAAATGC
670	CCCCCAAGCTTGGTGTACACTGTGAAGAACATCC
671	CGCGGATCCGGTAAACATGATGTACTTTACG

References

1. Sheff, M. A. & Thorn, K. S. Optimized cassettes for fluorescent protein tagging in Saccharomyces cerevisiae. *Yeast* **21**, 661-670 (2004).
2. Ragu, S. et al. Loss of the thioredoxin reductase Trr1 suppresses the genomic instability of peroxiredoxin tsa1 mutants. *PLoS One* **9**, e108123 (2014).
3. Sikorski, R. S. & Hieter, P. A system of shuttle vectors and yeast host strains designed for efficient manipulation of DNA in Saccharomyces cerevisiae. *Genetics* **122**, 19-27 (1989).
